# Control of Neutrophil Inflammation at Mucosal Surfaces by Secreted Epithelial Products

**DOI:** 10.3389/fimmu.2013.00220

**Published:** 2013-07-31

**Authors:** Rose L. Szabady, Beth A. McCormick

**Affiliations:** ^1^Department of Microbiology and Physiological Systems, University of Massachusetts Medical School, Worcester, MA, USA

**Keywords:** hepoxilin, eicosanoids, neutrophil migration, *Salmonella*, intestinal inflammation, lipoxygenase, MRP2, lipid chemoattractant

## Abstract

The human intestine is a large and delicately balanced organ, responsible for efficiently absorbing nutrients and selectively eliminating disease-causing pathogens. The gut architecture consists of a single layer of epithelial cells that forms a barrier against the food antigens and resident microbiota within the lumen. This barrier is augmented by a thick layer of mucus on the luminal side and an underlying lamina propria containing a resident population of immune cells. Attempted breaches of the intestinal barrier by pathogenic bacteria result in the rapid induction of a coordinated innate immune response that includes release of antimicrobial peptides, activation of pattern recognition receptors, and recruitment of various immune cells. In recent years, the role of epithelial cells in initiating this immune response has been increasingly appreciated. In particular, epithelial cells are responsible for the release of a variety of factors that attract neutrophils, the body’s trained bacterial killers. In this review we will highlight recent research that details a new understanding of how epithelial cells directionally secrete specific compounds at distinct stages of the inflammatory response in order to coordinate the immune response to intestinal microbes. In addition to their importance during the response to infection, evidence suggests that dysregulation of these pathways may contribute to pathologic inflammation during inflammatory bowel disease. Therefore, a continued understanding of the mechanisms by which epithelial cells control neutrophil migration into the intestine will have tremendous benefits in both the understanding of biological processes and the identification of potential therapeutic targets.

## Neutrophils: Critical Effectors of the Innate Immune Response

Most microbes encountered are non-pathogenic, and the human intestine is host to trillions of harmless or beneficial bacteria. The challenge for the gut immune system is to detect and defend against pathogenic invaders, while protecting commensals and host cells from a potentially damaging inflammatory response. Neutrophils are phagocytic innate immune cells that provide a first line of defense against bacterial infection [reviewed in (1)]. These cells are characterized by their polymorphic nuclei and short half-life, surviving approximately 9 h in circulation (2). Neutrophils are key components of the inflammatory response and are recruited following infection or sterile wounding. In addition to providing immune protection when barriers are breached, it has been suggested that neutrophils may contribute directly to resolution and recovery (3, 4). Genetic deficiencies in neutrophil function, such as mutations in the NADPH oxidase genes in patients with chronic granulomatous disease, result in severe immune defects and increased sensitivity to microbial infection (5). After migrating to effector sites, neutrophils kill pathogens in two distinct ways. Intracellular killing occurs by engulfment of bacteria and formation of a phagosome, which then fuses with intracellular granules to create a phagolysosome where microbes are killed by oxidative and non-oxidative mechanisms. Neutrophils also contribute to extracellular killing by discharging granular contents, releasing proteases, iron-binding proteins, defensins, and enzymes that catalyze formation of reactive oxygen and nitrogen species [reviewed in (1, 6)]. Additionally, neutrophils release “neutrophil extracellular traps” (NETs), structures made up of bacteria, histones, and attached granule enzymes that combine to disable and kill bacteria (7, 8). Once they have achieved their function, neutrophils die via apoptotic or non-apoptotic means and are cleared by macrophages during resolution of infection, although recent studies have challenged this paradigm of one-way neutrophil migration (9).

## Neutrophil Migration into the Intestine: Getting to the Site of Infection

Leukocytes migrate to their sites of activity via a carefully regulated multistep adhesion cascade [reviewed in (4, 10–12)]. Activated neutrophils first adhere to the endothelial cells that line the blood vessels, and then migrate across the endothelium and through the extracellular matrix to arrive at effector sites within the tissues. This occurs via a stepwise process consisting of tethering and rolling, activation, adhesion, and finally diapedesis. The exit sites for leukocyte emigration are the post-capillary venules, which are lined with endothelial cells expressing ligands that facilitate leukocyte adhesion. Neutrophils move through the bloodstream at a high flow rate, and the initial tethering process serves to slow the neutrophil’s movement and allow it to “roll” along the endothelial cell surface in order to sample for other potential signals. This tethering occurs via interaction of lectins and lectin receptors, and in the intestine is mediated primarily by the binding of endothelial P-selectin to neutrophil P-selectin glycoprotein ligand 1 (PSGL-1) (13–15). Rolling neutrophils become activated when they encounter endothelium-bound chemokines, leading to signaling through G protein-coupled receptors (GPCRs). This results in activation of β2 (CD18) integrins on the neutrophil surface, which interact with cell adhesion molecules (e.g., ICAM-1) and cause firm adhesion to the endothelium. This binding, though reversible, is stable for many hours and allows the cell to extravasate through the endothelial cell layer in order to reach its target site.

During inflammation of the intestinal mucosa, neutrophils that migrate across the endothelium and through the extracellular matrix to the base of the epithelial layer must undergo an additional transepithelial migration step in order to reach the lumen. Epithelial cells form a tight barrier whose permeability is regulated by the apical junction complex, which consists of proteins from adjacent cells interacting to form the tight junctions and adherens junctions [reviewed in (16)]. Crossing this barrier is necessary for neutrophils to defend against extracellular pathogens in the lumen, and also plays an important role in inflammatory pathology. Infiltration of neutrophils is associated with tissue damage at mucosal surfaces via mechanisms that include increased barrier permeability, epithelial apoptosis, and the release of damaging effectors such as reactive oxygen species and proteases (3, 17). Neutrophil accumulation on the basolateral side of the epithelium is insufficient to induce this pathology (18, 19), suggesting that the specific process of transepithelial migration is critical to the development of inflammatory pathology. Transepithelial migration shares some features with transendothelial migration, such as involvement of the CD11b/CD18 (Mac-1) integrin (20, 21), but also relies on unique interactions such as binding of CD47 and Sirp-α (22, 23). Meanwhile, neutrophil proteases such as elastase transiently degrade the epithelial tight junctions to permit movement across the paracellular space (24). Following migration, evidence suggests that specific ligands mediate neutrophil adherence to the apical surface before they actively detach in order to enter the lumen (25, 26). The importance of apical attachment of neutrophils is not fully understood, but it may be a strategy to encounter and destroy bacteria that are tightly attached to the epithelium. Indeed, apically attached PMN release inflammatory mediators that modulate epithelial responses (27). Apical attachment may also contribute to pathology during inflammation and promote the formation of crypt abscesses during inflammatory bowel disease (IBD) (25, 26, 28). Moreover, detachment of cells from the apical epithelium may be an important step in the resolution of neutrophilic inflammation (29).

## Recruitment of Neutrophils by Epithelial-Derived Chemokines

In addition to acting as the physical substrate for transepithelial migration, epithelial cells play a critical role in migration by producing chemotactic signals that recruit neutrophils out of the vasculature. CXC chemokines are the main class of chemoattractant ligands that recruit neutrophils and include CXCL1, CXCL2, CXCL5, CXCL6, and CXCL8, also known as IL-8, the prototypical neutrophil-attracting chemokine. These CXC chemokines bind to the two IL-8 receptors on the neutrophil surface, CXCR1 and CXCR2, with varying affinities and specificities (30). CXC chemokines can be released by a variety of cell types, including neutrophils themselves (31), but the prototypical pathway involves stimulation of epithelial cells resulting in secretion of IL-8. For example, infection of intestinal epithelial cells (IECs) with pathogenic bacteria results in flagellar stimulation of TLR5, followed by activation of NFkB signaling and upregulation of inflammatory pathways and subsequent IL-8 secretion (32, 33).

It is increasingly appreciated that neutrophils exhibit preferential attraction to specific molecules over others, a concept that has been critical to our understanding of how they can integrate and prioritize multiple chemoattractant gradients. Additionally, the “strength” of a specific chemoattractant to recruit neutrophils depends on the local environment (34). For example, IL-8 and the other CXC chemokines are sufficient to recruit neutrophils out of the vasculature, but once in the tissues cells will preferentially migrate toward leukotriene B_4_ (LTB_4_) or the C5a complement fragment. It seems therefore that IL-8 is an intermediate-stage chemoattractant, whereas C5a and LTB_4_ are “end-stage” chemoattractants that guide neutrophils to their final destination (35, 36). Formylated peptides such as fMLP are very strong neutrophil chemoattractants. It is thought that, because eukaryotic cells do not synthesize formyl peptides, these can serve as a unique bacterial signal to recruit neutrophils to sites of bacterial infection. Interestingly, mitochondria can also synthesize formylated peptides, possibly as a result of their early bacterial ancestry. Mitochondria-derived formyl peptides are released from dying cells and serve as damage associated molecular patterns (DAMPs) that can activate the immune response (37). Indeed, formylated peptides released by necrotic cells were found to serve as the critical end-stage chemoattractant during sterile inflammation (38). It is similarly possible that during inflammation, necrotic epithelial cells in the intestine release mitochondria-derived formyl peptides that attract neutrophils into the lumen, but this has not yet been demonstrated.

## Recruitment of Neutrophils by Bioactive Lipids

It had long been assumed that IL-8 production by epithelial cells was sufficient to drive neutrophil infiltration into the intestine. However, IL-8 undergoes polarized secretion from the basolateral surface of the epithelial cell and is too large to diffuse across the tight junctions into the lumen. *In vitro* findings confirm that although IL-8 is important for formation of a haptotactic gradient that guides neutrophils through the tissue to the basal epithelium, it is insufficient to drive the final transmigration step across the epithelial barrier (39, 40). In addition, mice overexpressing IL-8 specifically in IECs had increased recruitment of neutrophils to the epithelium, but in the absence of further inflammatory stimulus cells did not cross into the lumen or induce pathology (19), suggesting that an additional end-stage chemoattractant is required. This hypothesis was confirmed by the discovery that colonization of the epithelium by *Salmonella typhimurium* stimulates apical secretion of the eicosanoid hepoxilin A_3_ (HXA_3_), which then recruits neutrophils into the lumen (40, 41). Eicosanoids are bioactive lipids that are derived from arachidonic acid and mediate a variety of diverse cellular processes. Arachidonic acid is liberated from the cell membrane via the action of phospholipase A2 and converted into a variety of eicosanoids, including the pro-inflammatory neutrophil chemoattractants such as leukotrienes and hepoxilins as well as the anti-inflammatory lipoxins and resolvins (42). The type of eicosanoid that is produced depends on the activity of oxygenase enzymes with different specificities and sites of expression. For example, 5-lipoxygenase catalyzes the synthesis of pro-inflammatory leukotrienes and is expressed primarily in leukocytes, although there is some evidence that the 5-lipoxygenase dependent precursor, LTA_4_, can be processed transcellularly to generate active LTB_4_ (42, 43). Similarly, the anti-inflammatory lipoxins are generated by one of two transcellular mechanisms. LTA_4_ synthesized by 5-lipoxygenase in leukocytes can then serve as a substrate for adherent platelet-expressed 12-lipoxygenase, generating lipoxin A_4_ (LXA_4_) and LXB_4_. Alternatively, epithelial cells expressing 12-lipoxygenase generate 12-HETE from arachidonic acid, which is then acted on by leukocyte 5-lipoxygenase to generate LXA_4_ and LXB_4_ (44). Further work is needed to understand the signals that regulate this process and which pathways are functional during specific inflammatory and pro-resolving conditions.

## Hepoxilin A_3_ is an Essential Player in the Final Step of Neutrophil Migration into the Intestine

**Figure 1:**
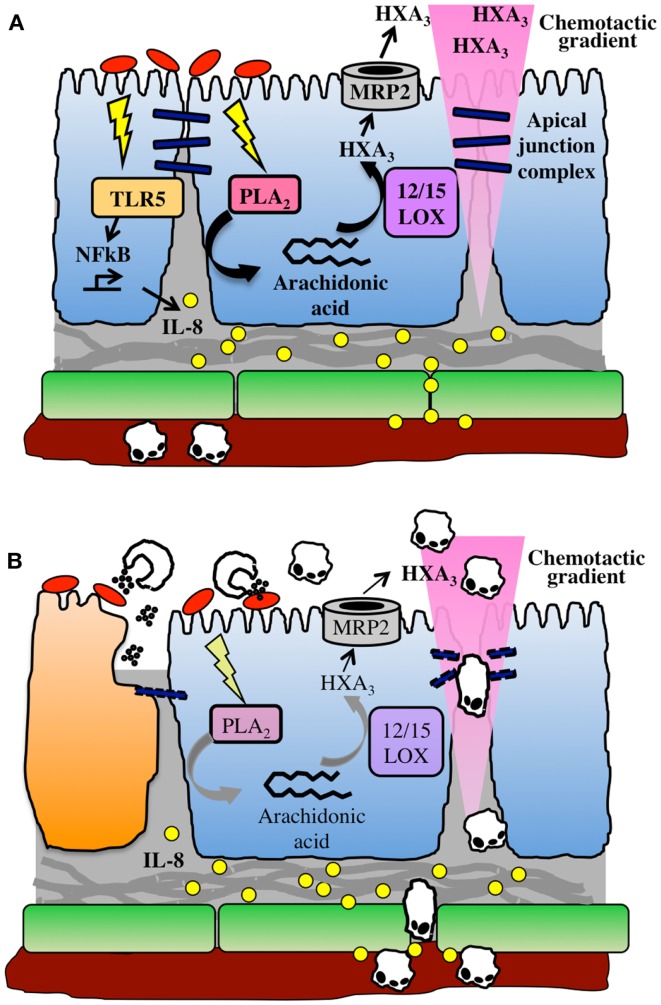
**The HXA_3_ inflammatory pathway**. **(A)** Infection of the epithelial cell (blue) surface by pathogenic bacteria (red) induces signaling through pattern recognition receptors, including TLR5, and activation of NFkB, leading to pro-inflammatory responses including basolateral secretion of IL-8. An IL-8 gradient forms that is imprinted in the subepithelial extracellular matrix, and IL-8 binds to endothelial cell (green) surface in order to recruit neutrophils out of the vasculature. Meanwhile, bacterial infection activates phospholipase A2-mediated liberation of arachidonic acid from the plasma membrane. Arachidonic acid is converted to HXA_3_ via the action of 12/15-lipoxygenase and secreted from the apical surface via the action of MRP2. HXA_3_ released into the lumen diffuses across the paracellular junction between epithelial cells to create a concentration gradient that will recruit neutrophils across the epithelium. **(B)** Neutrophils extravasate through endothelial cells into lamina propria, where they sense the HXA_3_ gradient, and migrate across the epithelial paracellular junction into the lumen. There, they encounter bacteria and release effectors including ROS and proteases, which can also lead to collateral damage to epithelial cells (dying cell shown in orange).

In contrast, HXA_3_ is formed directly in epithelial cells, from which it is secreted in order to create a chemoattractant gradient that drives neutrophils into the lumen. Arachidonic acid is first converted to 12-(S)HPETE by 12-lipoxygenase, and 12-(S)HPETE is then converted to HXA_3_ by hepoxilin synthase (45). Specific apical secretion of HXA_3_ is facilitated by multidrug resistant protein 2 (MRP2), an ATP-binding cassette transporter that is localized specifically to the apical surface of IECs (46). The extracellular HXA_3_ is freely diffusible across the paracellular space between epithelial cells and forms an apical to basolateral gradient (41). Neutrophils that have been recruited to the basolateral epithelium by IL-8 and other CXC chemokines can therefore sense the HXA_3_ gradient and transmigrate across the epithelium into the intestinal lumen. This can lead to host-protective clearance of bacteria during infection, but may also result in localized tissue destruction by neutrophil effectors (Figure 1).

Although many important features of the HXA_3_ biosynthetic pathway have been elucidated, intriguing questions remain. The HXA_3_ pathway was first identified as a response of epithelial cells to colonization with *S. typhimurium*, and was found to be essential for neutrophil transepithelial migration in this model (40, 41). Careful experiments identified SipA, a bacterial type III secreted protein, as an inducer of HXA_3_ secretion and showed that treatment with the purified protein alone was sufficient to induce neutrophil transepithelial migration (47, 48). Colonization with *S. typhimurium* leads to a SipA-dependent induction of HXA_3_ synthesis and concurrent upregulation of MRP2 (46). Further details of this pathway continue to be elucidated, including an important role for epithelial caspase-3 in processing SipA into its active form and the importance of both protein kinase C activity and ezrin activation in localizing MRP2 to the apical surface (49–51). Unlike IL-8 and fMLP, which also stimulate neutrophil activation, HXA_3_ seems to act as a pure chemoattractant for neutrophils in the absence of further activation (41). Binding of HXA_3_ to neutrophils induces calcium flux (52) and like other chemokine-receptor interactions, the signal is transduced through a pertussis toxin-sensitive GPCR (40).

While *S. typhimurium* infection of IECs is the best-studied model of HXA_3_ secretion, this pathway is in fact a far more universal mechanism by which epithelial cells respond to infection by driving inflammation and neutrophil recruitment. Other intestinal bacterial pathogens, including *Shigella flexneri* and enteroaggregative *Escherichia coli* O42 (EAEC), are also able to trigger 12-lipoxygenase and MRP2 dependent synthesis and secretion of HXA_3_ to drive neutrophil transepithelial migration (53–55). These bacteria do not possess homologs of SipA, and the critical bacterial effectors that activate HXA_3_ secretion remain to be identified. In the case of EAEC, it has been demonstrated that the aggregative adherence fimbriae (AAF) are necessary for the induction of HXA_3_-mediated neutrophil migration, and trans-expression of the AAF subunits was sufficient to confer an inflammatory phenotype on non-pathogenic *E. coli* (55). Interestingly, EAEC do not express a Type III secretion system, while *S. flexneri* do, but unlike *S. typhimurium* invade through the basolateral rather than the apical surface. These data suggest that divergent bacterial effectors must converge at some point leading to HXA_3_ synthesis and secretion, and the mechanisms underlying this convergence are an area of active investigation.

## The HXA_3_ Pathway also Drives Inflammation in the Lung Mucosa

Further evidence that HXA_3_ secretion is an important defense mechanism by which epithelial cells communicate to the immune system is evident from the discovery that this pathway is also functional in the lung mucosa. Bacterial pathogens including *Pseudomonas aeruginosa* and *Klebsiella pneumoniae* induce neutrophil transepithelial migration in an *in vitro* model of lung infection (56). Like *S. typhimurium*, *P. aeruginosa* induces IL-8 secretion from the basolateral side of the epithelium, and this IL-8 is similarly insufficient to drive migration across the epithelial layer. As in the intestine, bacterial infection of the lung epithelial cells triggers PKC activation, 12-lipoxygenase activity, and secretion of HXA_3_ (56, 57). The discovery of the shared reliance on apical secretion of HXA_3_ to drive the neutrophil inflammatory responses at different mucosal epithelial sites represents a paradigm shift in our understanding of how the epithelium controls the inflammatory response.

## A Potential Role for HXA_3_-Mediated Inflammation during Inflammatory Bowel Disease

Bacterial infection of the mucosal epithelium triggers a protective acute inflammatory response with neutrophil infiltration, and successful resolution of inflammation involves a dampening of the pro-inflammatory response and apoptotic clearance of inflammatory cells (3, 4). However, continuing pathologic inflammation is a hallmark of chronic inflammatory conditions such as IBD. While IBD is thought to result from the combination of an underlying genetic susceptibility and an environmental trigger, a specific bacterial pathogen has yet to be associated with the disease. More recently, it has been hypothesized that IBD may be associated with a “keystone pathogen,” a low abundance member of the microbiota that induces or promotes a dysbiotic state that results in inflammatory pathology (58). Patients with ulcerative colitis (UC) and Crohn’s disease exhibit specific pathology associated with continued damaging neutrophil infiltration, including crypt abscesses in Crohn’s and mucosal ulceration in UC (59). We have identified a role for the HXA_3_ pathway in mouse models of IBD as well as in association with human disease, suggesting that this pathway is a universal driver of intestinal inflammation beyond the acute response to infection with pathogenic bacteria. MRP2 expression at the epithelial surface is upregulated during chronic intestinal inflammation induced by CD45RBhi T cell transfer colitis in mice. Similarly, colonic biopsies from patients with active UC and Crohn’s disease demonstrate increased MRP2 staining at the epithelial surface (46). Furthermore, inhibition of 12-lipoxygenase activity with baicalin significantly reduced inflammatory pathology in T cell transfer colitis (46). These data suggest that HXA_3_ is in fact a critical driver of the continued infiltration of neutrophils into the intestine during colitis that leads to damaging inflammatory pathology.

A critical question, then, is what are the pathways that normally regulate HXA_3_-induced inflammation, and how do these pathways become dysregulated during chronic inflammation? It is increasingly appreciated that resolution of inflammation is not a passive process, but rather an active one that relies on specific pro-resolving mediators. This category includes a wide variety of lipid mediators derived from arachidonic acid or polyunsaturated fatty acids, including the lipoxins, resolvins, neuroprotectins, and maresins [reviewed in (44, 60)]. These lipids are largely synthesized by macrophages and monocytes that infiltrate during the resolution phase, some by way of a two-part transcellular biosynthesis as described above. It has not been conclusively demonstrated whether resolvins can be generated transcellularly or directly by mucosal epithelial cells. Several of these compounds, including LXA_4_ and resolvin D1, seem to act in opposition to the classical chemoattractant pathway by binding to GPCRs and transducing a stop signal for migratory cells (61, 62). It will be interesting to determine whether this stop signal is functional in the case of HXA_3_-mediated migration. *In vitro*, IECs express ChemR23, the receptor for resolvin E1 (RvE1), and RvE1 binding can induce anti-inflammatory effects (29, 63). It would be interesting to determine whether RvE1 also suppresses HXA_3_ biogenesis. Known anti-inflammatory cytokines such as IL-10 are other potentially interesting candidates. It is clear that a major area of continuing research interest lies in understanding how secretion of pro- and anti-inflammatory mediators by epithelial cells is regulated during inflammation. These studies will contribute to our further understanding of how the epithelium plays a critical role in initiating and sustaining the inflammatory immune response, with potential therapeutic implications for a wide variety of inflammatory conditions.

## Conflict of Interest Statement

The authors declare that the research was conducted in the absence of any commercial or financial relationships that could be construed as a potential conflict of interest.
